# Secretion of IFN-γ by Transgenic Mammary Epithelial Cells *in vitro* Reduced Mastitis Infection Risk in Goats

**DOI:** 10.3389/fvets.2022.898635

**Published:** 2022-06-24

**Authors:** Ying Liu, Hongyan Zhang, Shasha Dong, Boyu Li, Weiming Ma, Lijiang Ge, Zhiyong Hu, Feng Su

**Affiliations:** ^1^Shandong Provincial Key Laboratory of Animal Biotechnology and Disease Control and Prevention, College of Animal Science, Shandong Agricultural University, Taian, China; ^2^Department of Cardiology, The Second Affiliated Hospital of Shandong First Medical University, Taian, China

**Keywords:** IFN-γ, mastitis, gene-editing, anti-mastitis activity, infection risk

## Abstract

Mastitis results in great economic loss to the dairy goat industry. Many approaches have attempted to decrease the morbidity associated with this disease, and among these, transgenic strategy have been recognized as a potential approach. A previous mammalian study reports that interferon-gamma (IFN-γ) has potential anti-bacterial bioactivity against infection *in vitro*; however, its capacity *in vivo* is ambiguous. In this study, we initially constructed targeting and homologous recombination vectors (containing the IFN-γ gene) and then transferred the vectors into goat mammary gland epithelial cells (GMECs). Enzyme digestion and sequencing analysis indicated that the vectors used in this study were built correctly. Subsequently, monoclonal cells were selected using puromycin and the polymerase chain reaction (PCR) test indicated that IFN-γ was correctly inserted downstream of the casein promoter. Monoclonal cells were then assessed for reducible expression, and reverse transcriptase-PCR (RT-PCR) and Western blot tests confirmed that monoclonal cells could express IFN-γ. Finally, anti-bacterial capacity was evaluated using bacterial counts and flow cytometry analysis. Decreased bacterial counts and cell apoptosis rates in transgenic GMECs demonstrated that the secretion of IFN-γ could inhibit bacterial proliferation. Therefore, IFN-γ gene transfection in goat mammary epithelial cells could inhibit bacterial proliferation and reduce the risk of mammary gland infection in goats.

## Introduction

Mastitis is a complex disease caused by *Staphylococcus (S. aureus*), *Streptococcus* (*S. agalactiae*), and *Escherichia coli* (*E. coli*) infection; It can cause great economic loss to the dairy goat industry. There has been extensive research in this area, including dietary selection (1, 2), induction of immunity (3), and clinical treatments. However, none of these interventions have significantly decreased the incidence of mastitis. Recently, the development of transgenic animal strategies has supplied an effective way to reduce mastitis morbidity (4). Using this approach, Yuan et al. (5) and Liu et al. (6) produced anti-mastitis cattle by expanding the HBD3 gene. Furthermore, Liu et al. produced transgenic anti-mastitis cattle by transferring the human lysozyme gene (7), and Jun et al. developed mastitis resistant transgenic dairy goats and evaluated the levels of HBD3 secreted in the milk (8). These experiments had no damaging effects on the environment (9). Moreover, this series of experiments provided an effective means of producing mastitis resistant goats. During these studies, one important step was to evaluate the anti-mastitis capacity of potential genes that could be manipulated during breeding, especially in host cells. Thus, choosing an anti-mastitis gene and evaluating its anti-bacterial capability in GMECs was an important step in producing genetically modified goats.

Interferon-gamma (IFN-γ) is a remarkably small molecule that is used in clinical treatments as it participates in immune response and resistance against infections (10). For mastitis, IFN-γ can potentially be used to reduce bacterial proliferation in cases of mastitis. In *Staphylococcus* infection, IFN-γ is generated from the capsular polysaccharide simulated T cells and boosts the injurious effects of resistant *staphylococcus* (9). In *Streptococcal* mammary infection, proliferative *Streptococcus* is inhibited by the highly-expressed IFN-γ that is simulated by the *Streptococcus* (11). Meanwhile, during *E. coli* infection, IFN-γ was first used to control acute bovine mastitis during the periparturient period (12). Application of IFN-γ may elicit functional changes on other lymphocytes and phagocytic cells in the mammary gland, which can reduce the levels of mastitis (13). In this way, milk secretion from mammary cells is decreased and the phagocytic capacity of macrophages is increased, which in turn reduces mastitis morbidity. Thus, IFN-γ is recognized as a pivotal target site for developing mastitis resistance in goats. However, the anti-bacterial capacity of secreted IFN-γ in mammary gland epithelial cells has not been described and needs further evaluation before the production of genetically modified goats.

In this study, the anti-mastitis capability of human IFN-γ was evaluated in IFN-γ-inserted transgenic GMECs. To do this, we transferred the IFN-γ gene into GMECs using the CRISPR/Cas 9 system and assessed the secretory ability of resultant positive cells. Subsequently, anti-bacterial capacity was evaluated by using bacterial resistance assays.

## Materials and Methods

### Ethics Statement

The lactating dairy goats used in this study were purchased from Zhengda Company, Taian, China, and were accommodated in appropriate livestock housing and fed *ad-libitum*. Mammary tissue samples were obtained from three 20-month-old pregnant goats. Laoshan dairy goats were anesthetized with sodium barbital, then the mammary samples were collected during surgery in a sterile environment. All procedures were approved by the Animal Care and Use Committee of Shandong Agricultural University.

### Vector Construction

The gene target LentiCRISPRv2 (Cat: 52961, Addgene, Cambridge, MA) vector and homologous recombination vector (pCDH-CMV-MCS-EF1-CopGFP-T2A-Puro) were both purchased from Addgene. The sgRNAs were designed from the website http://crispr.mit.edu/ and then inserted into the LentiCRISPRv2 vector after the vector was digested by *Bsmb* I (Abcam Company, Cambridge, UK). The casein promoter (LA: long arm) was amplified into the PMD-18T vector (Takara Company, China), then the IFN-γ gene sequence (NM_000619), synthesized by the Shanghai Shenggong Company (Shanghai, China), was inserted behind the casein promoter using overlap polymerase chain reaction (PCR). The LA-IFN-γ sequence was inserted into the homologous recombination vector after double digestion by the *Spe* I (Takara Company, China) and *EcoR* I (Takara Company, China) enzymes. Subsequently, the short arm (SA) was amplified and inserted into the recombination vector after digestion by the *Kpn* I (Takara Company, China) enzyme.

### Preparation of Goat Mammary Epithelial Cells (GMECs)

Mammary epithelial tissue was collected from Laoshan dairy goats. Tissues were washed thrice with PBS, and minced into several pieces of around 1 mm^3^. Tissue blocks were placed into 60 mm petri dishes with Dulbecco's Modified Eagle Medium (DMEM)/F12 [containing 10% fetal bovine serum (FBS) and 10 ng/mL epidermal growth factor (EGF: Beyotime, Shanghai, China)] in a cell incubator at 37°C and 5% CO_2_. The medium was refreshed every 2 days.

### Virus Package, Cell Transfection, and Single Clone Cell Selection

The recombination vectors and target vectors were packaged separately within human 293T cells. Human 293T cells were cultured into 100-mm cell culture dishes, the target vector (LentiCRISPRv2, 7.5 μg), homologous recombination vector (7.5 μg), with package vectors psPAX2 (6 μg, Addgene, #12260) and pCMV-VSV-G (6 μg, Addgene, #8454) were co-transfected into human 293T cells to produce lentiviral particles. Cell media was collected after co-infection with 293T cells at 24 and 48 h and then followed by a 0.45 μm filtering (Millex^®^-HV). The concentrated virus was obtained after mixing with PEG8000 reagent overnight followed by centrifugation. The virus was then resuspended with fresh DMEM/F12 medium and used to infect the isolated GMECs. The cells were selected using puromycin (1 μg/ml) over a period of 3 weeks and monoclonal cells were identified. The monoclonal cells were separated and individually transferred into a new dish to proliferate for future assays.

### PCR and RT-PCR Assays

The monoclonal cells were proliferated in a 60-mm dish in a cell incubator at 37°C and 5% CO_2_. Subsequently, the cells were divided into three aliquots, one of which was for RNA extraction and reverse transcription. The primers that were used for insert site detection were designed at both sides of the IFN-γ expression cassette. A PCR assay was used to assess IFN-γ insertion after the DNA was extracted using a DNA extraction kit (DNAzol Reagent, Cat: 10503027). Reverse transcriptase-PCR (RT-PCR) primers were used to assess IFN-γ expression in transgenic GMECs. All the primers used in this study are listed in Table 1.

**Table 1:** The primers that used in this study.

**Name**	**Sequence (5'-3')**	**Length (bp)**	**Annealing temperature (**°**C)**	**Application**
SA-F	AGCAGGTACCGTCTAAGAGGATTTC	729	52	Short arm
SA-R	TGCAGGTACCTAACTCTTCATCTCAC			
LA-I-F	ATCACTAGTTGGGGACTGGGCAAGAGAA	926	60	Long arm
LA-I-R	TACATATGGGTCCTGTGCAATGGCCAGAGCCACCAGACAGGCA			
A-IFN-F	TGGTGGCTCTGGCCATTGCACAGGACCCATATGTAAAAGA	1,358	60	Arm + IFN
A-IFN-R	GAGCGAATTCTTACTGGGATGCTCTTCG			
KY1-F	TGCAATAATATCCTCCCT	1,652	56	Integration site detection
KY1-R	CGACAGTTCAGCCATCAC			
KY2-F	AAATCCTGGTTGCTGTCT	1,603	54	Integration site detection
KY2-R	GCCTAAGGGTTAATTTATTG			
hx2-F	ACCTTGGCCATATGATAAG	600	55	Digestion efficiency
hx2-R	TCTTGTTGGTCTGTTGCT			
hx8-F	AGAAGAAACTTATTGGGA	600	54	Digestion efficiency
hx8-R	AAATCTGTCAACACCATA			
IFN-F	CAGGACCCATATGTAAAAGAAG	429	60	RT-PCR
IFN-R	TTACTGGGATGCTCTTCGAC			

### Inducible Expression

The primary cells (non-transgenic GMECs) and monoclonal cells (transgenic GMECs) were separately cultured into 90-mm dishes. When the cells reached 80% confluence, the inductive medium [Opti-MEM with 10 ng/mL EGF, 1% ITS Liquid Media Supplement, 5 ng/mL prolactin (Sigma, cat: L6520), and 1 mg/mL hydrocortisone] was replaced with the previous medium. The supernatants were then collected at 24 h and concentrated for Western blot analysis.

### Western Blot Analysis

Concentrated proteins were used for Western blot analysis following a standard protocol. Briefly, the proteins were transferred onto a PVDF (polyvinylidene-fluoride) membrane after protein separation by SDS-PAGE electrophoresis. The membrane was then incubated with IFN-γ antibody (LMAI Bio, Shanghai, China) at a 1:1000 dilution after blocking with a blocking buffer for 4 h. Subsequently, the membrane was incubated with HRP goat anti-rabbit IgG (Beyotime Institute of Biotechnology, Shanghai, China) at a 1:2000 dilution for 2 h. The membrane was then exposed after being treated with a chemiluminescence substrate according to the manufacturer's instructions. All experiments were repeated independently three times.

### Bacterial Infection Assay

All bacteria used in this assay were purchased from the Chinese Institute of Veterinary Drug Control (Beijing, China) and preserved in the lab. *E. coli* (ATCC25922), *S. aureus* (ATCC25923), and *S. agalactiae* (ATCC12386) were cultured at 37°C in 100 mL trypticase soy broth medium until the optical density (OD 600) value was ≥1. Subsequently, the bacteria were centrifuged at 3,000 *g* (acceleration of gravity) for 5 min followed by three washes and finally re-suspending in phosphate buffered saline (PBS) at 1 × 10^7^ colony forming units (CFU)/mL. GMECs were counted and cultured in 12-well dishes, then cultured in DMEM/F12 overnight. After inducible expression of IFN-γ as mentioned above, the cells were then infected with these bacteria separately at a multiplicity of infection (MOI) of 10:1. Cells and their supernatants were collected separately after 2 h and used for other experiments. Cells were used for flow cytometry analysis and bacterial count evaluation at 6 h. The risk of anti-bacterial infection was evaluated by bacteria colony-forming unit (CFU) counts in GMECs (14). All experiments were repeated independently three times.

### Flow Cytometry

The infected cells were stained using Annexin V PE/7-AAD (Qiagen, Valencia, CA, USA). Cell apoptosis and death rates were then evaluated using flow cytometry analysis.

### Statistical Analysis

Data from the CFU count assay were analyzed using SPSS software (SPSS, Chicago, IL, USA). The CFU data are means ± SD and were compared using one-way ANOVA followed by the Newman-Keuls test. *P*-values < 0.05 were considered statistically significant.

## Results

### Inducible Secretion of IFN-γ From GMECs Was Controlled by a Transgenic Strategy

**Figure 1:**
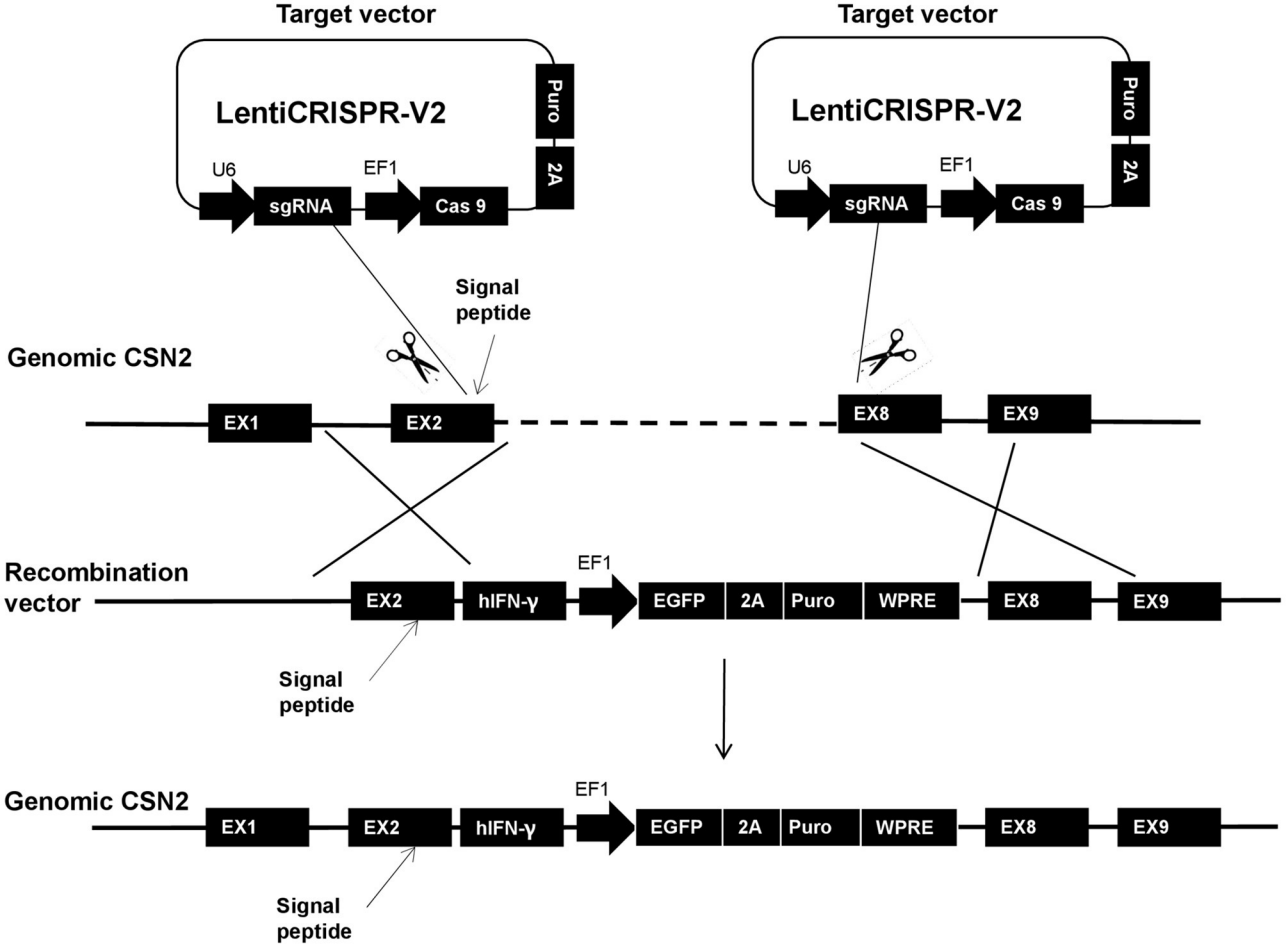
The entire homologous integration scheme of this research. In the strategy, one target site was chosen in the 2nd exon of the casein (CSN2) gene, upstream of the signal peptide of the gene. The other site was located upstream of the 8th exon. The homologous arm (long arm: LA) of the recombination vector was designed containing the CSN2 signal peptide and used for guiding interferon-gamma (IFN-γ) secretion.

Transgenic GMECs were processed using the CRISPR/Cas 9 system; the detailed scheme is shown in Figure 1. From the scheme, one of the target sites was chosen in the 2nd exon of the casein (CSN2) gene, upstream of the signal peptide of the gene. The other one was located upstream of the 8th exon. The homologous arm (LA) of the recombination vector was designed containing the CSN2 signal peptide sequence and used to guide IFN-γ secretion.

### Targeted and Homologous Combination Vector Construction

**Figure 2:**
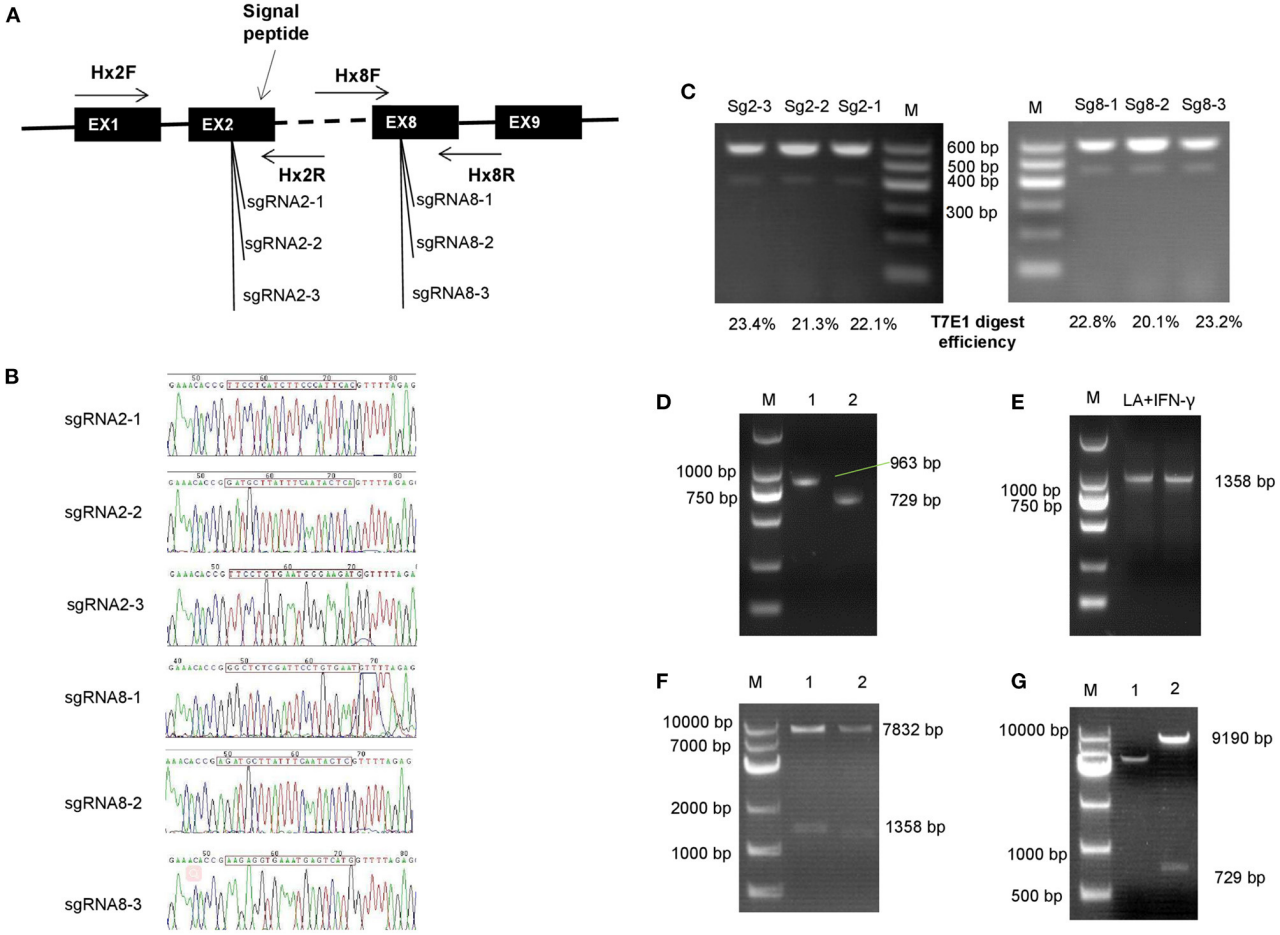
Construction of gene-targeted and homologous integration vectors. **(A)** Location of single guide RNAs (sgRNAs) that were used in this study. **(B)** Sequencing of the target vector; the sgRNAs were inserted into the Lenti-CRISPR V2 vector followed by sequencing. **(C)** Digestion efficiency analysis of sgRNAs. **(D–G)** Homologous recombination vector construction process. **(D)** The homologous arm was amplified and assessed by polymerase chain reaction (PCR) assay. The bands in line 1 and lane 2 independently represent the long and the short arm length. **(E)** Interferon-gamma (IFN-γ) was synthesized and inserted downstream of the long arm by overlap PCR, which was about 1,358 bp in length. **(F)** Long arm inserting assay, the vector was double digested by *Spe* I and *EcoR* I enzymes. Two bands in lane 1 and 2 showed that the long arm with IFN-γ was completely inserted into the homologous vector. **(G)** Enzyme identification of the entire combination vector. The short arm was digested from the recombination vector by *Kpn* I.

Gene targeted vectors were built based on the Lenti-CRISPR V2 system; the single guide RNAs (sgRNAs) were designed on the 2nd and the 8th exon, separately (Figure 2A). Accuracy of sgRNA nucleotides were evaluated by sequencing; the sequence, shown in Figure 2B, shows that the target vectors were built correctly. Subsequently, the digestion efficiency of sgRNAs was assessed by PCR assays after the DNA was cut by the T7E1 enzyme. Shearing efficiency of Sg2-3 and Sg8-3 was higher than for other sgRNAs; this is shown in Figure 2C. Figures 2D–G show the homologous recombination vector construction process. Figure 2D shows the homologous arm amplification. The bands in lane 1 and lane 2 represent the separate LA and SA lengths. Subsequently, IFN-γ was synthesized and inserted downstream of the LA using overlap PCR, which was about 1,358 bp in length (Figure 2E). Figure 2F shows the LA inserting test, two bands in lanes 1 and 2 show that the LA with IFN-γ is completely attached to the homologous vector. Finally, the SA was correctly inserted into the recombination vector after sequencing (Figure 2G).

### Inducible Secreted IFN-γ in Gene-Edited GMECs

**Figure 3:**
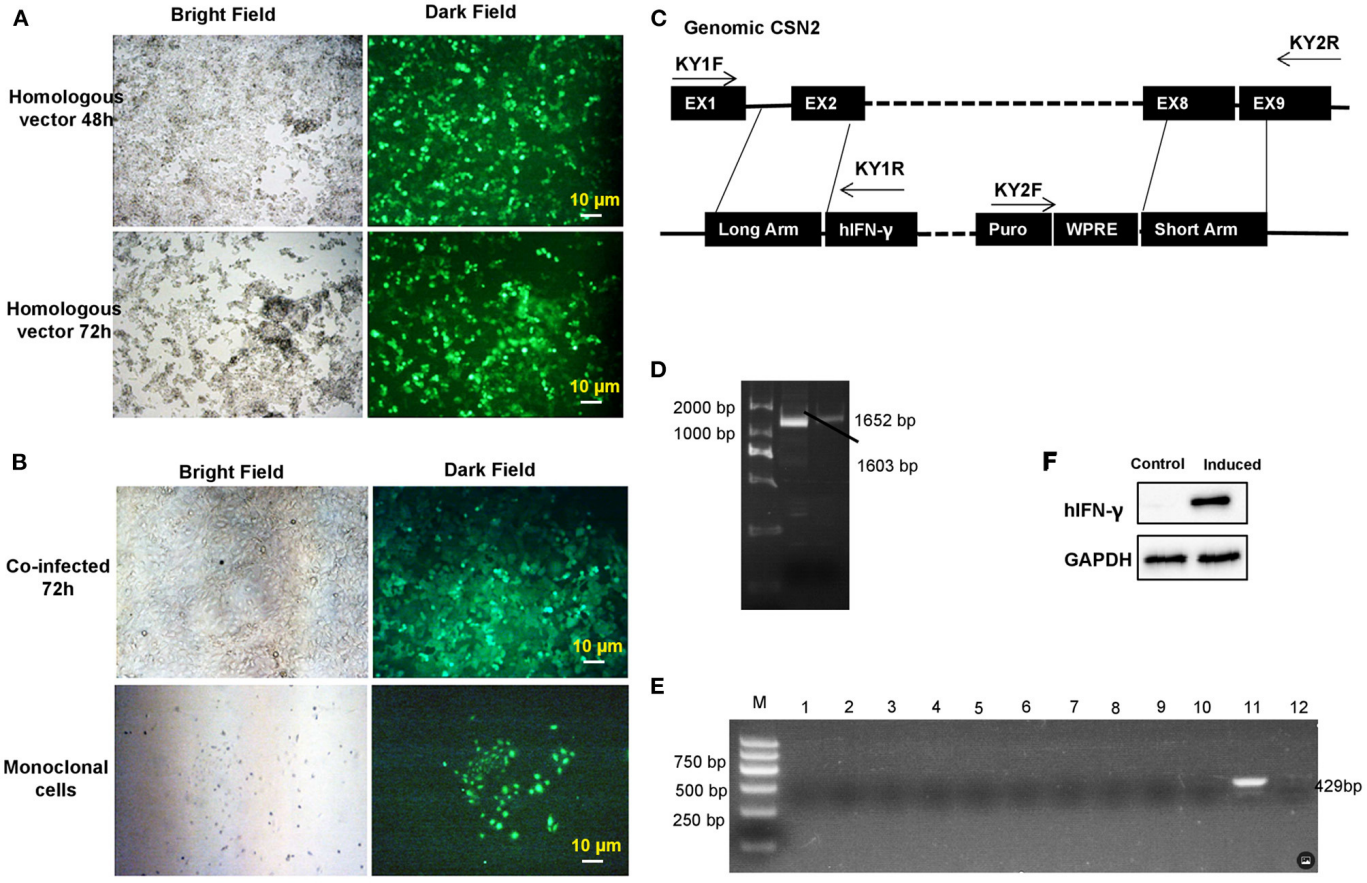
Transgenic monoclonal goat mammary gland epithelial cells (GMECs) screening and evaluation. **(A)** Lentivirus plasmids were packaged in 293T cells. Homologous and target vectors were all packaged in 293T cells. Expression of green fluorescent protein (GFP) in 293T cells confirmed that the infection rate of homologous vectors was sufficient for the virus concentration. **(B)** The monoclonal GMECs that were obtained after puromycin selection. **(C,D)** Integrated interferon-gamma (IFN-γ) expression cassettes were evaluated by PCR assays. Inserted site detection indicated the expression cassettes were correctly integrated into the genome of GMECs. Middle lane **(D)** in the figure is the KY2 PCR product, the right lane **(D)** is KY1 PCR product. **(E)** Detection of IFN-γ mRNA in the monoclonal cells by RT-PCR after inducible expression. **(F)** Western blot analysis of inducible IFN-γ. Analysis of IFN-γ indicated that the gene-edited GMECs could secrete IFN-γ protein as expected.

Transgenic GMECs were obtained after co-infection of target and homologous recombination vectors in 293T cells. Expression of green fluorescent protein (GFP) in 293T cells confirmed that the infection rate of these vectors was efficient for the packaged virus (Figure 3A). Monoclonal GMECs were obtained following selection with puromycin (Figure 3B). Integrated IFN-γ expression cassettes were then evaluated by PCR tests. Inserted site detection indicated that the expression cassette was correctly integrated into the GMEC genome (Figures 3C,D). Subsequently, all monoclonal cells were induced and IFN-γ mRNA proteins were evaluated by RT-PCR and Western blot, respectively. RT-PCR assays in GEMCs indicated that the 11^th^ monoclonal cells could express IFN-γ mRNA (Figure 3E). Finally, Western blot analysis of IFN-γ indicated that the gene-edited GMECs could secrete IFN-γ protein as expected (Figure 3F).

### Gene-Edited GMECs Displayed Strong Bacterial Resistance

**Figure 4:**
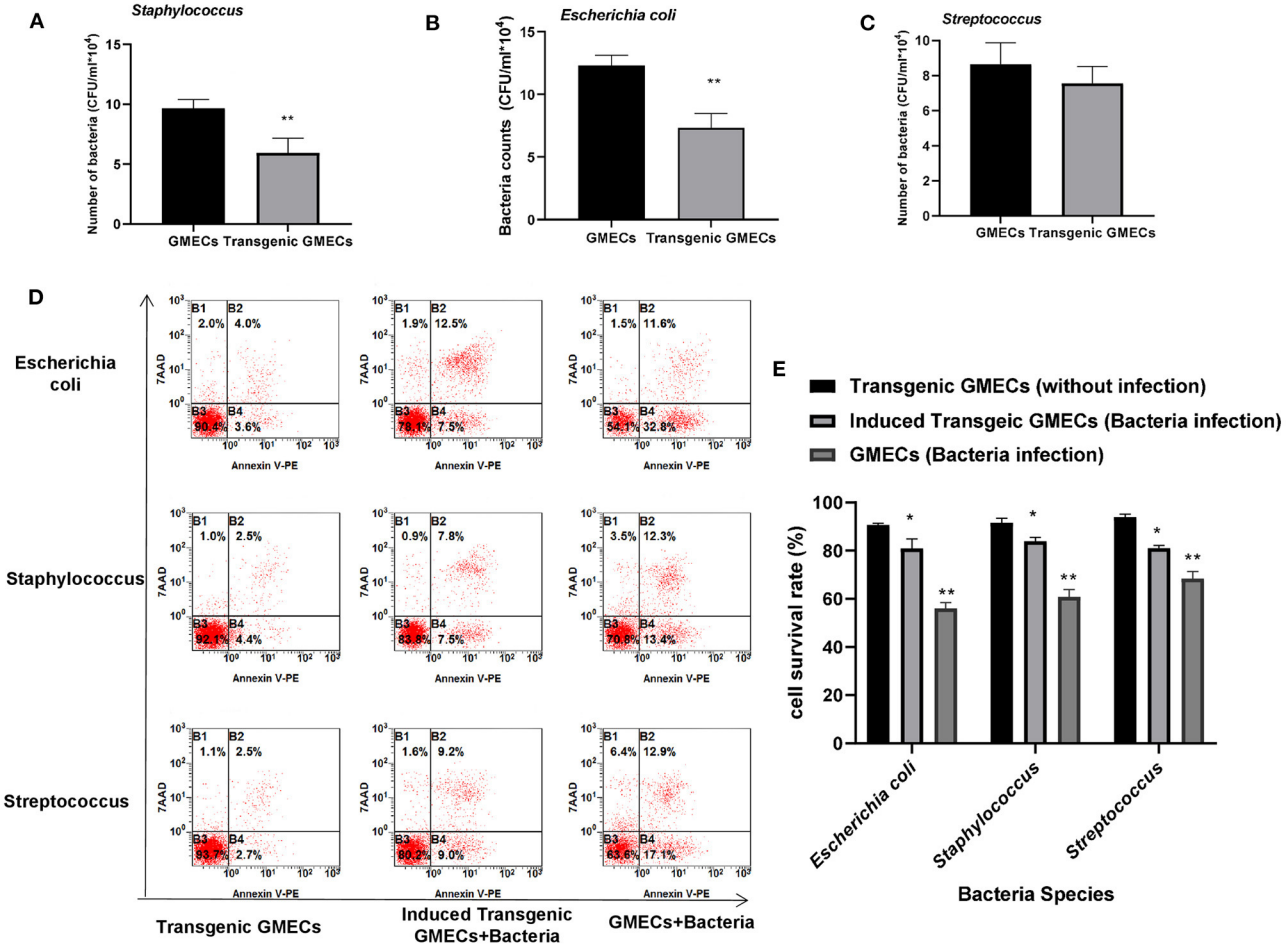
Anti-bacterial capacity of transgenic monoclonal goat mammary gland epithelial cells (GMECs) that expressed and secreted interferon-gamma (IFN-γ) protein. **(A–C)** Bacterial challenge of transgenic GMECs was performed to assess the anti-bacterial capacity of IFN-γ. Transgenic GMECs showed much stronger *Staphylococcus* and *Escherichia coli* resistant activity than the non-transgenic ones (*P* < 0.01), but no obvious differences in anti-*Streptococcus* effects. **(D,E)** Survival rate of GMECs in different bacteria-treated cells at 6 h; transgenic GMECs obviously increased the survival rate over non-transgenic ones (*P* < 0.05). *p < 0.05, **p < 0.01.

Bacterial challenge of transgenic GMECs was performed to assess the anti-bacterial capacity of IFN-γ. Transgenic GMECs showed much stronger *Staphylococcus* and *E. coli* resistance activity than the non-transgenic ones (*P* < 0.01), but no obvious differences in anti-*Streptococcus* effects (Figures 4A–C). GMEC survival rate in different bacteria-treated cells showed that the transgenic GMECs had an obviously increased survival rate over non-transgenic ones (Figures 4D,E).

## Discussion

Mastitis can be economically devastating to the dairy goat industry. Many approaches have been investigated to decrease potential mastitis morbidity, with the transgenic strategy being recognized as a crucial development. A previous study reported that IFN-γ has the potential to increase resistance to bacterial infection in mammalian cells *in vitro*. However, the anti-bacterial capacity of IFN-γ *in vivo* is still ambiguous (11, 13). In this study, we first constructed gene targeting and homologous recombination vectors and transferred these vectors into GMECs. Subsequently, monoclonal cells were selected and then used for IFN-γ reducible expression. Finally, anti-bacterial effects were evaluated, and the decreased bacterial counts and cell apoptosis ratio in transgenic GMECs demonstrated that the secretion of IFN-γ could inhibit bacterial proliferation and decrease the risk of mammary infection.

Accurate nucleotide editing technology is an effective method for decreasing the morbidity of animal diseases (15). Regarding bovine mastitis, Liu et al. generated anti-mastitis cows *via* zinc-finger nickase-mediated insertion of the HBD3 (human-beta-defensin 3) gene (6) and lysostaphin (7). All transgenic cows had increased anti-bacterial levels and mastitis resistance. In his studies, casein (CSN2) promoter was used to drive HBD3/lysostaphin expression and the signal peptide of CSN2 was used to guide protein secretion (7). In the current study, expression and secretion of IFN-γ was also driven by the CSN2 promoter, as previously reported. The difference between these studies is mainly focused on the cut points. Liu's study used one cut point in the CSN2 promoter while the current study used two main points, one of which was located upstream of the signal peptide and the other one located in exon 8 of the CSN2 gene. The existence of exon 2 and exon 8 in CSN2 may contribute to the stable secretion of IFN-γ.

Target rates of sgRNAs were evaluated by DNA-cleaving rates. The target rate was not as high as previously reported: This may be attributed to species difference (16) as Zhang's laboratory used human cells as targets (17) while this study only used goat cells.

Transgenic GMECs were selected and the expression of IFN-γ was assessed by Western blot analysis. Expression of the GFP protein indicated that the monoclonal cells that were screened had a high purity, as previously reported (9). Homologous arm detection and inducible expression assays confirmed IFN-γ could be expressed correctly in 11th monoclonal cells. The main reason for this appearance maybe relevant to the methylation effect of the CSN2 promoter (18).

A previous study reported that IFN-γ could activate NADPH oxidase, enhance killing of ingested *Staphylococcus* by phagocytosis, and increase the binding of *Staphylococcus*-neutrophil complexes to macrophages (19). It has also been shown that *E. coli* can induce IFN-γ expression which increases bacterial phagocytosis through modulation of macrophage oxidant status (20). In the current study, anti-microbial capacity was assessed by bacterial counts and target cell apoptosis rates. Decreased *Staphylococcus* and *E. coli* counts in IFN-γ secreted transgenic GMECs indicated that IFN-γ could inhibit *Staphylococcus* and *E. coli* proliferation by killing bacteria directly or indirectly, which agrees with previous studies (9). The survival rates of *Staphylococcus* and *E. coli* infected transgenic GMECs also support this finding. In *Streptococcus* infected transgenic GMECs, secretion of IFN-γ is insufficient to inhibit *Streptococcal* proliferation (21), though the survival rates of transgenic GMECs were obviously higher than those of non-transgenic ones (11). The main reason for this difference may be attributed to the potential immune function. A previous study reports that IFN-γ plays important roles in *Streptococcus* control by regulating macrophage activation, immune cell recruitment, leukocyte activation, and other aspects of innate specific immunity in different pathways (22). On the one hand, inducible expressed IFN-γ could activate GMEC immune reaction to defend against *Streptococcal* infection which then decreases streptococcal counts. However, GMECs protected against bacterial invasion rather than activated the immune system as an immune reaction was unable to be activated by *Streptococcus* because of the deficiency of CD4^+^ receptors in GMECs. A previous study reported B group *Streptococcus* (including *S. agalactiae*) could produce IFN-γ in CD4^+^ T cells in *in vivo* or *vitro* models (23). A deficiency of CD4^+^ receptors in GMECs is the main reason for the lack of IFN-γ and an ineffective inhibition of *Streptococcal* duplication. On the other hand, there is the bacterial susceptibility of GMECs. Günther's study revealed that *S. uberis* failed to activate an immune reaction in mammary epithelial cells (24). Ariffin's assays report that *S. agalactiae* was isolated from dairy goat milk (25) but there is still no direct evidence confirming the infection capacity of this bacteria. In addition, the T cells in *S. uberis* may have simulated the immune reaction *in vitro*.

The production of genetically modified dairy goats is an effective scheme for mastitis resistance. However, the first step is to verify the effect of the inserted gene. In this study, the anti-mastitis capacity was evaluated using the anti-bacterial level of human IFN-γ in genetically modified GMECs. This assay confirmed that IFN-γ can be used to improve goats' resistance to mastitis, especially when breeding transgenic animals. Nevertheless, there are some drawbacks in the assays used that need further exploration, such as the anti-bacterial mechanisms of human IFN-γ in different bacteria.

In conclusion, this study constructed gene target and homologous recombination vectors and then transferred IFN-γ into GMECs. The transgenic GMECs were then selected and monoclonal GMECs were used to induce the secretion of IFN-γ. Expression of IFN-γ in transgenic GMECs showed a strong anti-bacterial capacity; in particular, it decreased *Staphylococcus* and *E. coli* bacterial counts. Cell survival rates in transgenic GMECs confirmed that secreted IFN-γ in transgenic GMECs obviously reduced apoptosis, which in turn decreased the risk of mastitis. Therefore, IFN-γ could potentially become a preventive treatment leading to the reduction of mastitis in dairy goats through the use of gene editing.

## Data Availability Statement

The original contributions presented in the study are included in the article/supplementary material, further inquiries can be directed to the corresponding authors.

## Ethics Statement

The animal study was reviewed and approved by Animal Care and Use Committee of Shandong Agricultural University. Written informed consent was obtained from the owners for the participation of their animals in this study.

## Author Contributions

YL and FS designed the experiment and drafted the manuscript. HZ, YL, and SD carried out the animal care, samples collection, and performed the experiments. BL and LG performed the data processing and biological information analysis. FS and WM interpretation of data. ZH and FS conceived the study and writing the manuscript. All authors read and approved the final manuscript.

## Funding

This research was financially supported by grants from National Key R&D Program of China (Grant Number: 2021YFD1200902) and Shandong Natural Science Foundation (Grant Numbers: ZR2016CQ25 and ZR2016CQ29).

## Conflict of Interest

The authors declare that the research was conducted in the absence of any commercial or financial relationships that could be construed as a potential conflict of interest.

## Publisher's Note

All claims expressed in this article are solely those of the authors and do not necessarily represent those of their affiliated organizations, or those of the publisher, the editors and the reviewers. Any product that may be evaluated in this article, or claim that may be made by its manufacturer, is not guaranteed or endorsed by the publisher.

## Abbreviations

IFN-γ: interferon-gamma
GMECs: goat mammary gland epithelial cells
*S. aureus*: *Staphylococcus*
*S. agalactiae*: *Streptococcus*
*E. coli*: *Escherichia coli*..
